# Effect of Short-Anchored PEGylated Lipids on Lipid Nanoparticle Characterization Profiles, Stability, and Efficacy

**DOI:** 10.3390/biomedicines14051002

**Published:** 2026-04-28

**Authors:** Caroline E. R. Souleyrette, Phillip C. West, Stacy S. Kirkpatrick, Joshua D. Arnold, Michael R. Buckley, Michael B. Freeman, Oscar H. Grandas, Lauren B. Grimsley, Michael M. McNally, Deidra J. H. Mountain

**Affiliations:** Department of Surgery, Division of Vascular and Endovascular Surgery, University of Tennessee Health Science Center College of Medicine-Knoxville, 1924 Alcoa Highway Box U-11, Knoxville, TN 37920, USA; csouleyrette@utmck.edu (C.E.R.S.); pwest@utmck.edu (P.C.W.); skirkpat@utmck.edu (S.S.K.); jarnold@utmck.edu (J.D.A.); mbuckley@utmck.edu (M.R.B.); mfreeman@utmck.edu (M.B.F.); ograndas@utmck.edu (O.H.G.); lbgrimsley@utmck.edu (L.B.G.); mmcnally@utmck.edu (M.M.M.)

**Keywords:** drug delivery, lipid nanoparticles, gene therapy, RNA interference, PEGylation, cellular uptake, gene silencing, nanoparticle stability, critical quality attributes

## Abstract

**Background/Objectives**: RNA interference (RNAi) is a promising strategy for mitigating diseases at the molecular level. However, RNAi is limited by its instability in biological fluids and impermeability to cellular membranes. In response, our lab has previously patented a non-ionizable lipid nanoparticle (LNP) platform (R8-PLP) for RNAi therapeutic delivery. This formulation incorporates 1,2-distearoyl-sn-glycero-3-phosphoethanolamine-N-[methoxy(polyethylene glycol)-2000] (DSPE-PEG) to improve particle stability and drug retention. However, long-anchored PEGylated lipids like DSPE-PEG may impair internalization and stimulate immune responses. The literature suggests substituting short-anchored PEGylated-lipids like 1,2-dimyristoyl-rac-glycero-3-[methoxy(polyethylene glycol)-2000] (DMG-PEG) to attenuate these effects. Here, we evaluated whether substituting DMG-PEG for DSPE-PEG in our R8-PLP would improve in vitro cellular delivery and gene transfection without compromising in vitro critical quality attributes (CQAs) or increasing cytotoxicity. **Methods**: CQAs [encapsulation efficiency (EE%), particle size (nm), homogeneity (polydispersity index; PDI), and membrane zeta-potential] were assessed at assembly and after storage for up to 28 days at 4 °C. Additionally, in-serum stability at 4 °C and serum release kinetics at 37 °C were assessed. Human aortic smooth muscle cells (HASMCs) were treated with R8-PLPs and analyzed for cellular uptake (fluorometry), cytotoxicity (LIVE/DEAD stain), and gene modulation (qPCR). **Results**: DMG-PEG incorporation at variable mol% did not alter R8-PLP size, homogeneity, or siRNA EE% at assembly or after long-term storage, but did accelerate siRNA release kinetic profiles compared to DSPE-PEG controls. DMG-PEG substitution enhanced cellular uptake compared to DSPE-PEG R8-PLPs without increasing cytotoxicity. DMG-PEG incorporation also achieved significant silencing versus non-treated controls but did not improve gene silencing compared to DSPE-PEG R8-PLPs. **Conclusions**: Thus, DMG-PEG substitution did not enhance R8-PLP in vitro gene modulation efficacy despite improving cellular uptake and maintaining CQAs.

## 1. Introduction

Ribonucleic acid (RNA) therapeutics are a promising strategy for mitigating diseases at the molecular level. RNA interference (RNAi) is the most advanced RNA therapeutic technology and the closest to being broadly clinically translated. However, RNAi is limited by its instability in biological fluids and impermeability to cellular membranes [1]. Therefore, naked RNAi is inherently inefficient and must be paired with a carrier for functional delivery to achieve therapeutic efficacy.

A wide range of RNAi delivery strategies have been developed, many of which utilize nanoparticle-based complexes. These systems typically rely on electrostatic interactions between positively charged carrier components and the negatively charged phosphate backbone of RNAi molecules [2,3]. Some common classes include cationic polymers (i.e., chitosan, star polycations, and guanidinium-functionalized polymers), peptide-based systems (i.e., cell penetrating peptides and branched amphiphilic peptide capsules), inorganic nanoparticles (i.e., layered double hydroxides), organic nanoparticles (i.e., carbon quantum dots), and lipid-based nanoparticles [2,3,4]. While many of these are under active investigation for RNAi development, lipid-based nanoparticles are the most advanced and clinically validated therapeutic delivery technology to date, capable of protecting RNAi cargo from nuclease degradation while simultaneously facilitating internalization. Within this category, lipid nanoparticles (LNPs) are widely regarded as an ideal delivery vehicle relative to other platforms due to their biocompatibility, low toxicity, versatility, and potential for targetability [5]. One of the primary components of LNPs is polyethylene glycol (PEG), a polymer incorporated to improve LNP stability and pharmacokinetics [6].

PEG is a hydrophilic molecule that, when conjugated to a lipophilic molecule, protrudes from the surface of LNPs to create a steric barrier [6]. This barrier inhibits particle aggregation and increases circulation half-life by preventing opsonization and phagocytosis by the mononuclear phagocytic system (MPS) [7]. Though advantageous, this physical hindrance can also impair LNP efficacy by mitigating surface to surface interactions necessary for initiating target cell uptake and endosomal escape, a paradox known as the PEG Dilemma [1,6]. Moreover, PEGylation can inhibit LNP efficacy by inducing an immune response resulting in the production of anti-PEG antibodies [6,8]. As a result of this antibody presence, secondary administrations of PEGylated therapies are often ineffective due to their rapid clearance [7,8]. Thus, PEGylation can attenuate LNP internalization and long-term efficacy.

The current literature suggests that PEG’s unfavorable attributes can be influenced by the length of its hydrophobic lipid anchor. Specifically, shorter lipid tails weakly anchor the PEG molecule in the LNP membrane, resulting in its spontaneous dissociation in serum [9,10,11]. This limited retention exposes the LNP surface, permitting membrane interactions necessary for internalization without sacrificing PEG-mediated LNP stability. Likewise, because free PEG is immunogenically inert [12,13], this rapid dissociation reduces antibody production and subsequent LNP clearance [10,14,15]. As a result, LNPs that incorporate short-tailed PEGylated lipid moieties have demonstrated improved cellular uptake, enhanced gene silencing, and attenuated immune activation compared to long-tailed PEGylated lipids [10,14,16].

Our group has previously developed and patented a non-ionizable naturally derived LNP platform (R8-PLP) capable of efficacious packaging and delivery of RNAi therapeutics [17,18]. Our current R8-PLP technology was developed for the delivery of gene therapeutics to vascular tissues, specifically targeting post-surgical secondary intimal hyperplasia induced restenosis, the primary clinical focus of our laboratory. This R8-PLP technology utilizes a long 18-carbon tailed PEGylated lipid 1,2-distearoyl-sn-glycero-3-phosphoethanolamine-N-[methoxy(polyethylene glycol)-2000] (DSPE-PEG) for membrane stability and load retention. However, in light of the current literature and PEG’s potential deleterious effects, we hypothesize that alternatively incorporating a short 14-carbon tailed PEGylated lipid 1,2-dimyristoyl-rac-glycero-3-[methoxy(polyethylene glycol)-2000] (DMG-PEG) could further enhance the R8-PLP platform. Specifically, we investigated if substitution of DSPE-PEG with DMG-PEG, at variable mole percents, would enhance R8-PLP cellular uptake and gene silencing in vitro in vascular cell types, without increasing cytotoxicity or sacrificing nanoparticle critical quality attributes (CQAs).

## 2. Materials and Methods

### 2.1. LNP Formulation

#### 2.1.1. LNP Constituents

All lipid nanoparticle formula components are defined in Table 1. Lipids and cholesterol were purchased from Avanti Polar Lipids, Inc. (Alabaster, AL, USA). Stearylated octaarginine was purchased from LifeTein LLC (Somerset, NJ, USA).

#### 2.1.2. LNP Assembly

Base R8-PLPs were formulated with bulk lipid DOPC and Chol at a mole ratio of 7:3 plus 10 mol% STR-R8 and 10 mol% DSPE-PEG to serve as the experimental control. This standard R8-PLP platform was modified via the substitution of DMG-PEG for DSPE-PEG, at variable 1, 5 and 10 mol% as the experimental groups. The control and experimental formulation constituents and their mol% incorporation are detailed in Table 2. We denote the individual components of our formulations as mole parts of 100 total mole, with each component having their respective mole percent (mol%). These values, along with their respective molecular weights, are used to calculate the volume of each constituent at assembly. Reductions in mol% PEG content were adjusted for by increasing the DOPC and Chol content, while still maintain a 7:3 mole ratio DOPC-Chol as our bulk membrane constituent throughout. Because this primary bulk proportion is consistent across formulations, whereby minimizing significant compositional shifts, the primary variable under investigation presents as the degree of PEGylation across groups. All LNPs were assembled using a previously established EtOH injection technique with a Lipid: small-interfering RNA (siRNA) weight-to-weight ratio of 100:1. Briefly, lipids were dissolved in CHCl_3_, combined as indicated and dried under N_2_ gas and vacuum to remove remaining solvent. Dried lipids were then resuspended in molecular grade 100% EtOH and injected dropwise into 10 mM Tris-HCL, pH 8.0 with 10 mM CaCl_2_ and either *GAPDH* siRNA for targeted gene silencing or CDH1 siRNA as a nonfunctional negative control gene target (Thermofisher Scientific, Waltham, MA, USA). Injections were performed at a 2:3 EtOH:aqueous volume ratio, under constant vortexing at room temperature. LNPs were then purified from EtOH via 24 h dialysis against phosphate-buffered saline (PBS), pH 7.4 at 4 °C and extruded using a 100 nm polycarbonate membrane TWIST mini extruder prior to cellular assays (Helix Biotech, Knoxville, TN, USA).

### 2.2. LNP CQA Characterization Studies

#### 2.2.1. siRNA Encapsulation Efficiency

The siRNA encapsulation and retention of all LNP formulations were measured using the Quant-it RiboGreen RNA Reagent (Thermofisher Scientific). After dialysis purification, LNPs were denatured with 1% Triton X-100 and 100 µg/mL heparin at 37 °C for 15 min to release their siRNA cargo. Released siRNA was mixed with RiboGreen reagent for fluorescent labeling and the emission was read at 525 nm. Fluorescence units of the denatured LNPs was fit to a known standard curve of siRNA in 1% Triton X-100 and 100 µg/mL heparin. The encapsulation efficiency (EE%) of each LNP formulation was calculated as (pmols siRNA encapsulate/total original pmols siRNA added) × 100.

Additionally, the RiboGreen Assay was used to test each LNP formulation without the addition of 1% Triton X-100 and 100 µg/mL heparin to assess the LNP membrane for drug retention and/or permeability. Fluorescent units of the intact LNPs were then measured to detect any siRNA in the surrounding solution.

#### 2.2.2. Size, Homogeneity, and Zeta-Potential

The mean LNP size (nm), associated LNP homogeneity (polydispersity index, PDI), and membrane zeta-potential (mV) were measured post-dialysis in PBS by dynamic light scattering (DLS) using the Zetasizer Nano ZS instrument (Malvern Instruments Ltd., Worcestershire, UK).

#### 2.2.3. Transmission Electron Microscopy for Qualitative LNP Morphology

R8-PLPs were applied on carbon-coated copper grids, washed briefly in distilled water, stained with 1% *w*/*v* uranyl acetate in water, and blotted with Whatman #1 filter paper. After complete drying, samples were observed with a JEOL 1400-Flash Transmission Electron Microscope (JEOL USA, Inc., Peabody, MA, USA) at 120 kV acceleration voltage. Images were acquired using a Gatan OneView camera (Gatan, Inc., Pleasanton, CA, USA).

#### 2.2.4. LNP Long-Term Stability in PBS

R8-PLPs were assembled as described at 100:1 Lipid:siRNA with variable mol% DMG-PEG, dialyzed overnight and stored in PBS diluent at 4 °C for up to one month. The siRNA retention, mean LNP size, and associated homogeneity (PDI) were assayed for changes over time, as described above, as a marker of LNP membrane stability under readily attainable clinical storage conditions. The LNP characterization profiles were assessed after dialysis as a baseline (Day 0) and again at 1, 4, 7, 14, 21, and 28 days post-storage at 4 °C.

#### 2.2.5. LNP Stability in Serum

R8-PLPs were assembled as described at 100:1 Lipid:siRNA with variable mol% DMG-PEG, dialyzed overnight and stored in 10% fetal bovine serum (FBS) diluted in PBS at 4 °C for up to 7 days. The siRNA retention, mean LNP size, and associated homogeneity (PDI) were assayed for changes over time, as described above, as a marker of LNP membrane stability in serum. The LNP characterization profiles were assessed after dialysis as a baseline (Day 0) and again at 1, 4, and 7 days post-storage at 4 °C.

#### 2.2.6. LNP Drug Release Kinetics

R8-PLPs were assembled as described at 100:1 Lipid:siRNA with variable mol% DMG-PEG, dialyzed overnight and incubated in 0% and 20% FBS diluted in PBS at 37 °C for up to 18 h. Ribogreen assay was used to measure the release of siRNA cargo over time at 0-, 6-, 12- and 18 h as an indicator of in-serum release kinetics and LNP membrane stability under physiological conditions. Percent siRNA release was normalized to total initial siRNA encapsulate of each formulation at assembly time 0.

### 2.3. In Vitro Cellular Assessments

#### 2.3.1. Vascular Smooth Muscle Cell Culture

Human aortic smooth muscle cells (HASMCs) were obtained from LifeLine Cell Technology LLC (Frederick, MD, USA) as cryopreserved primary cell cultures isolates from a 17 yr old male donor. HASMCs have been selected as our primary cell line for LNP testing and development to date, due to their involvement in vascular pathogenesis, the ultimate clinical target of our LNP delivery system’s development. Cells were incubated at 37 °C in environmental conditions of 5% CO_2_ and 95% humidity and grown in VascuLife growth medium (VascuLife Basal Medium + VascuLife smooth muscle cell supplement kit + gentamycin/amphotericin; Lifeline Cell Technology).

#### 2.3.2. Cellular Uptake Studies

To measure cellular uptake, LNPs were assembled as described with the addition of Rho-PE at 1 mol%. HASMCs were grown to ~80% confluency, serum-starved for 24 h in Dulbecco’s Modified Eagle Medium (DMEM; Thermofisher Scientific) to induce a quiescent state, then treated for 24 h with Rhodamine-labeled LNP formulations at 50 µM total lipid in Vasculife growth medium. After 24 h LNP exposure, cells were washed 3 times with 1× filtered PBS.

Qualitative cellular LNP uptake was assayed by fluorescent microscopy. Cells were fixed with 10% phosphate buffered formalin for 15 min prior to imaging. Images were obtained on a BX51 Olympus microscope (Olympus Q-color camera, Olympus Corporation, Shinjuku, Tokyo, Japan) at 100× with a Texas Red fluorescent filter.

For quantitative uptake analysis, cells were trypsinized and pelleted, lysed with 1% Triton X-100 and centrifuged at 12,000 RPM for 5 min to remove cell debris. Cell lysates were plated in duplicate and measured by fluorometry at 575 nm on a Modulus Microplate Multimode Reader (Promega Corporation, Madison, WI, USA). Cellular uptake was quantified by the average fluorescent units of each duplicate, minus the average baseline fluorescence of nontreated control cells. Experiments were repeated with n = 3 and each replicate normalized to its own replicate control of R8-PLP 10% DSPE-PEG.

#### 2.3.3. Cytotoxicity Analysis

To measure LNP-induced cytotoxicity, HASMCs were grown to ~60% confluency, serum-starved for 24 h, then treated for 24 h with LNP formulations at 50 µM total lipid in DMEM. Cell toxicity was quantified using LIVE/DEAD^®^ Viability/Cytotoxicity Kit (Thermofisher Scientific), according to the manufacturer’s instructions. Briefly, cell media was removed, cells were washed three times in PBS and co-stained with calcein-AM + ethidium homodimer for 15 min at 37 °C. Stained cells were visualized via fluorescein isothiocyanate (FITC; live) and Texas Red (dead) fluorescent filters. Images were acquired in triplicate on the BX51 Olympus microscope for both filters at 100×. Cells were counted using ImagePro Premier Version 9.2 software (Media Cybernetics, Inc., Rockville, MD, USA). Percent cellular toxicity was calculated as [dead cell count/(dead cell count + live cell count)] × 100 in each image and triplicate images were averaged per independent sample.

#### 2.3.4. In Vitro Gene Silencing

To assay LNP-mediated silencing efficacy, LNPs were assembled as described and loaded with a targeted *GAPDH* siRNA cargo. Although our long-term goal for this LNP technology is modulation of disease-relevant gene targets for vascular disease mitigation, many of these gene targets are not constitutively expressed in healthy unstimulated HASMC cultures. Thus, here we selected a highly expressed housekeeping gene (i.e., *GAPDH*) as a proof-of-concept target due to its abundant expression and ease of efficacy evaluation. HASMCs were grown to ~60% confluency, serum-starved in DMEM for 24 h, then treated with LNP formulations at 50 nM siRNA in DMEM. After 24 h treatment, cells were washed 3 times with 1× filtered PBS and grown for an additional 24 h in Vasculife growth medium prior to collection for quantitative polymerase chain reaction (qPCR). Total RNA was isolated using the Invitrogen PARIS Kit Protein and RNA Isolation System (Thermofisher Scientific) according to the manufacturer’s instructions, and 250 ng of RNA isolate was converted into cDNA using the High-Capacity RNA-to-cDNA Kit (Thermofisher Scientific). Two microliters of cDNA were then amplified in duplicate by qPCR using the TaqMan Gene Expression Master Mix and predesigned TaqMan Gene Expression Assays specific for human *GAPDH* on the StepOne PCR system (Applied Biosystems, Foster City, CA, USA). The relative quantity of *GAPDH* messenger RNA (mRNA) was determined using the comparative cycle threshold method, with 18S ribosomal RNA as an endogenous control, in duplicate. Percent gene expression for each LNP formulation was normalized to the relative *GAPDH* expression in non-treated controls.

### 2.4. Statistical Analysis

All data are reported as mean ± standard error of the mean. Statistical analyses were performed using either Student’s *t*-test or One-Way with Tukey post hoc pairwise comparison, as appropriate. Probability (*p*) values <0.05 were considered significant.

## 3. Results

### 3.1. Substitution of DMG-PEG for DSPE-PEG Results in Slight Morphological Distinctions in R8-PLPs, but Does Not Alter Their LNP Structure

**Figure 1:**
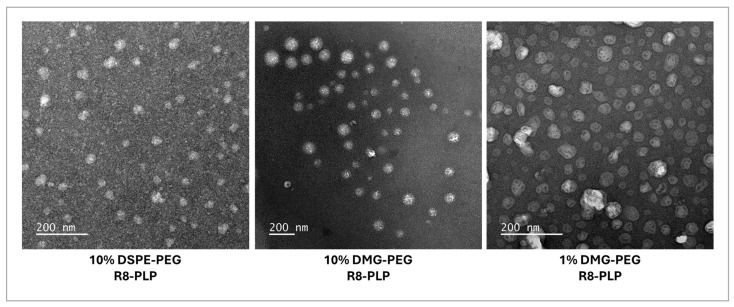
Conventional TEM images of R8-PLP. All formulations form nanoscale, predominantly spherical particles with variable internal contrast and no visible bilayer structures, consistent with LNP-type assemblies. Differences between formulations are observed primarily in particle compactness and electron density.

Conventional transmission electron microscopy (TEM) revealed LNP-like morphology for all three formulations, characterized by roughly spherical particles with unstructured internal contrast and no distinct bilayer structures (Figure 1). Qualitative observations indicate that both PEG lipid composition and PEG anchor chemistry influence LNP compactness and qualitative morphology, but without changing their distinct nanoparticle structural classification.

### 3.2. Substitution of DMG-PEG for DSPE-PEG Does Not Affect Total siRNA Encapsulation Efficiency or Modify R8-PLP Characterization Profiles at Assembly

**Figure 2:**
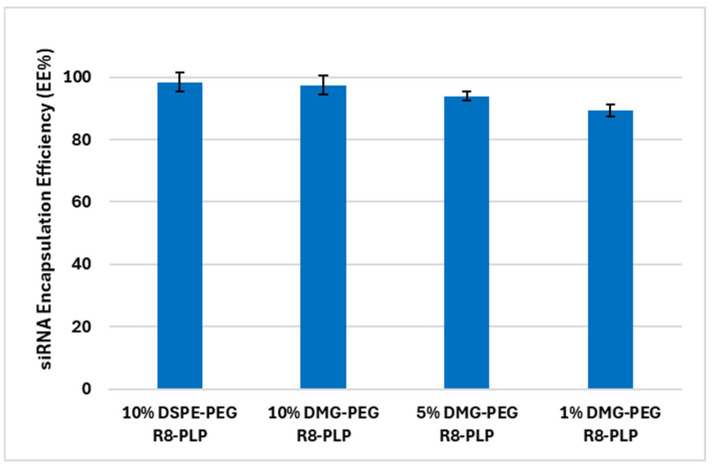
Effect of variable mol% DMG-PEG on R8-PLP cargo loading efficiency. Substitution with DMG-PEG did not affect siRNA EE% when compared to R8-PLP 10% DSPE-PEG control. P = NS, n = 3.

R8-PLPs formulated with 10, 5 and 1 mol% DMG-PEG resulted in 97.46 ± 3.0%, 93.87 ± 1.4%, and 89.35 ± 2.0% total siRNA EE%, respectively (Figure 2), and was not significantly different than total EE% in the R8-PLP 10% DSPE-PEG controls (98.44 ± 3.1%). Furthermore, encapsulation in all groups was around or better than 90%, an in vitro CQA considered best-in-class and a necessary preliminary criterion for prospective clinical realization (Figure 2). Additionally, DLS of R8-PLPs formulated with variable mol% DMG-PEG demonstrated favorable LNP sizes (<65 nm) and narrow size distribution (<0.3 polydispersity index, PDI) at assembly (Table 3), CQAs comparable to the R8-PLP 10% DSPE-PEG controls. Likewise, the membrane charge (zeta-potential) of R8-PLP 10% DMG-PEG was not different compared to equivalent R8-PLP 10% DSPE-PEG controls, both well below the +10 mV threshold (Table 3). As expected, membrane zeta-potential increases as % DMG-PEG is reduced; however, all groups remained <+20 mV at assembly.

### 3.3. Substitution of DMG-PEG for DSPE-PEG Does Not Affect R8-PLP Drug Retention or Characterization Profiles After Long-Term Storage in PBS at 4 °C for up to One Month

**Figure 3:**
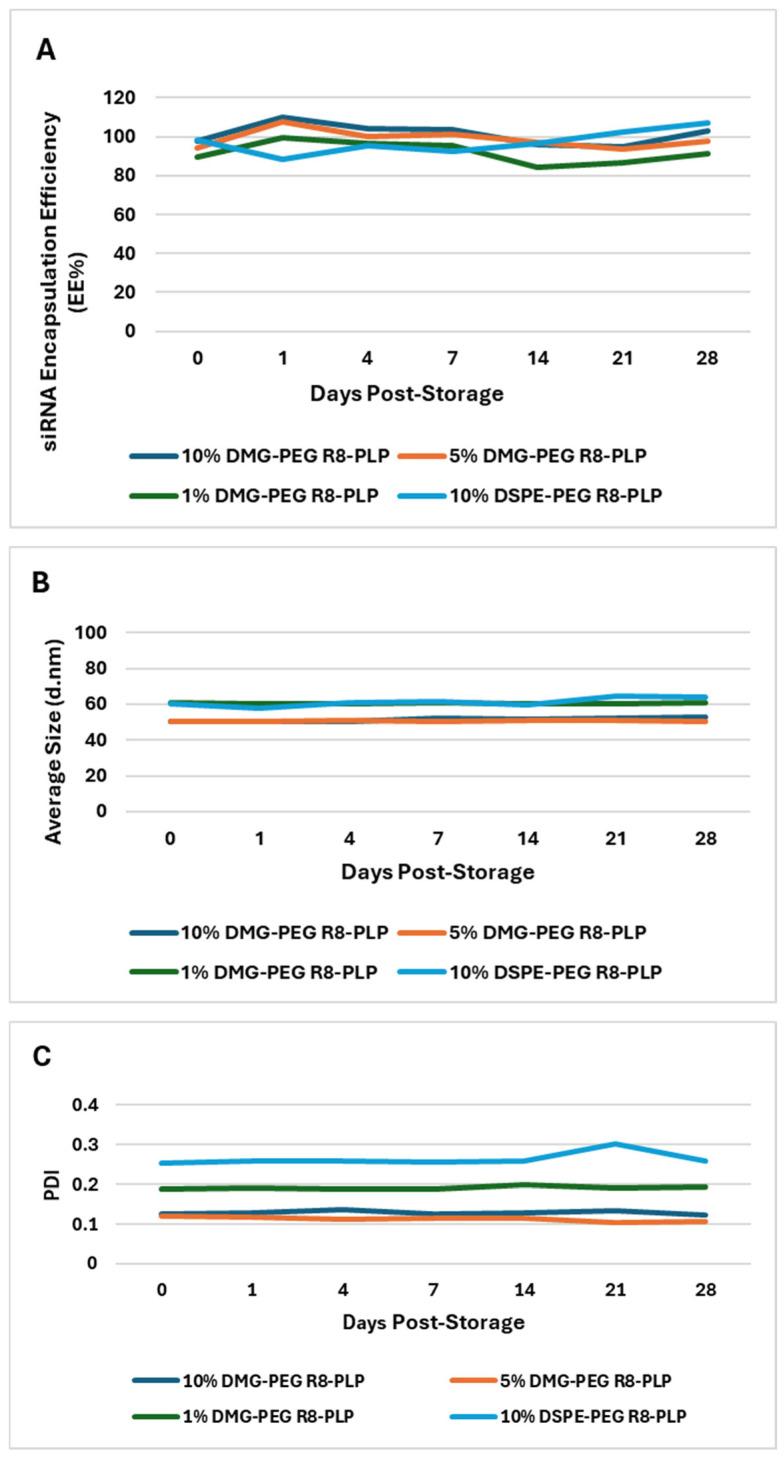
Effect of variable mol% DMG-PEG on R8-PLP in vitro CQAs and LNP long-term stability and storage. Incorporation of DMG-PEG did not affect R8-PLP characterization profiles at assembly or upon storage at 4 °C for up to 28 days. All R8-PLP formulations with variable mol% DMG-PEG displayed no change in (**A**) siRNA EE%, (**B**) average particle size (d.nm), or (**C**) homogeneity (PDI) over time when compared to their baseline profile at assembly (day 0). *p* = NS vs. Day 0, n = 3.

Encapsulation efficiency, drug retention, size and PDI of each R8-PLP formulation were assessed at assembly (day 0) and serially following storage at 4 °C for up to 28 days. R8-PLPs formulated with 10, 5 and 1 mol% DMG-PEG, as well as 10 mol% DSPE-PEG controls, demonstrate full siRNA load retention (EE% ≥ 85%) for up to 28 days and no change in size or PDI when compared to their baseline assembly profile (day 0) (Figure 3, Figure S1.).

### 3.4. Substitution of DMG-PEG for DSPE-PEG Does Not Affect R8-PLP In-Serum Stability or siRNA Drug Retention After Storage in Serum at 4 °C for up to One Week

**Figure 4:**
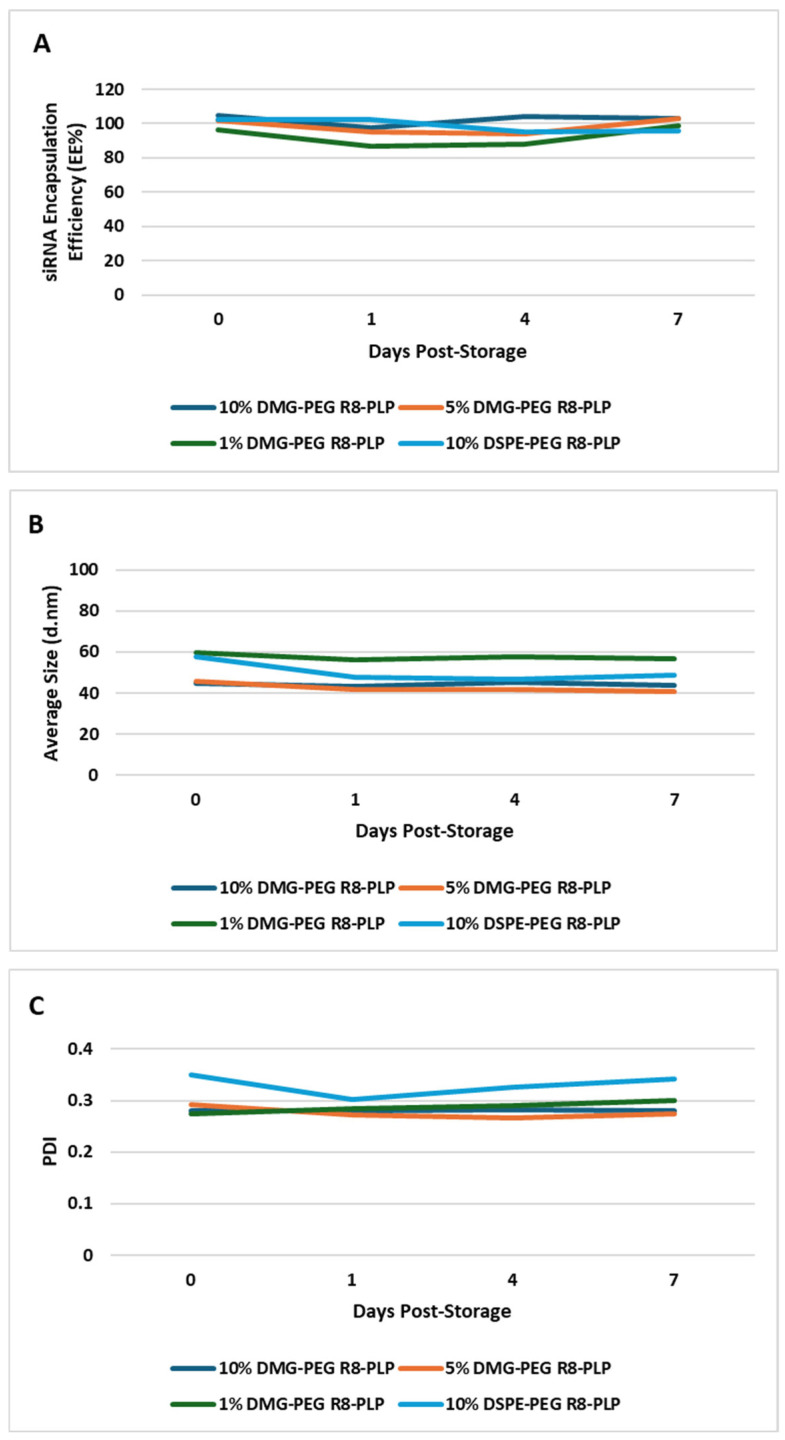
Effect of variable mol% DMG-PEG on R8-PLP in-serum stability. Incorporation of DMG-PEG did not affect R8-PLP characterization profiles after storage in serum at 4 °C for up to 7 days. All R8-PLP formulations with variable mol% DMG-PEG displayed no change in (**A**) siRNA EE%, (**B**) average particle size (d.nm), or (**C**) homogeneity (PDI) over time when compared to their baseline profile at assembly (day 0). *p* = NS vs. Day 0, n = 3.

Encapsulation efficiency, drug retention, size and PDI of each R8-PLP formulation were assessed at assembly (day 0) and serially following storage in serum at 4 °C for up to 7 days. R8-PLPs formulated with 10, 5 and 1 mol% DMG-PEG, as well as 10 mol% DSPE-PEG controls, demonstrate full siRNA load retention (EE% ≥ 85%) for up to 7 days and no change in size or PDI when compared to their baseline assembly profile (day 0) (Figure 4).

### 3.5. Substitution of DMG-PEG for DSPE-PEG Accelerates R8-PLP siRNA Release Kinetics in Serum at 37 °C

**Figure 5:**
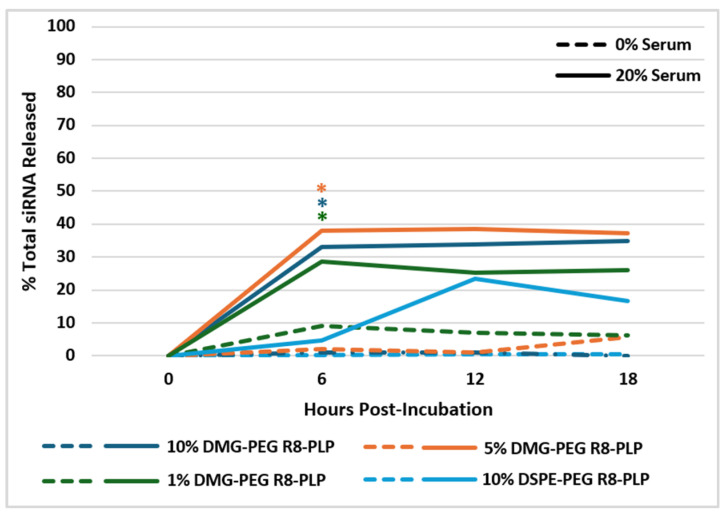
Effect of variable mol% DMG-PEG on R8-PLP siRNA release kinetics in serum at 37 °C. Incorporation of DMG-PEG did not affect R8-PLP siRNA drug retention profiles upon incubation in 0% serum at 37 °C for up to 18 h. All DMG-PEG R8-PLPs demonstrated significant siRNA release as early as 6 h in 20% serum, when compared to DSPE-PEG R8-PLP controls. * *p* < 0.05 vs. 20% serum 10% DSPE-PEG R8-PLP Hour 6, n = 3.

Total siRNA encapsulate of each R8-PLP formulation was assessed at assembly (Hour 0) and the % of total siRNA released was measured serially for up to 18 h with incubation in 0% and 20% serum at 37 °C. R8-PLPs formulated with 10, 5 and 1 mol% DMG-PEG, as well as 10 mol% DSPE-PEG controls, demonstrate minimal siRNA release of <10% total encapsulate in 0% serum. In contrast, in 20% serum 10, 5, and 1 mol% DMG-PEG R8-PLPs demonstrated significantly accelerated siRNA release as early as 6 h (33.0 ± 5.78%, 38.0 ± 5.86%, and 28.6 ± 5.72%, respectively) when compared to 10% DSPE-PEG R8-PLP controls at 6 h (4.5 ± 3.97%, Figure 5, * *p* < 0.05, n = 3), suggesting that in the absence of a cell-based environment, DMG-PEG desorption may lead to greater serum-mediated membrane susceptibility via temperature-enhanced serum–protein interactions. Comparatively, DSPE-PEG R8-PLP controls exhibited comparable release only after 12 h compared to baseline at assembly hour 0 (Figure 5).

### 3.6. Substitution of DMG-PEG for DSPE-PEG Enhanced R8-PLP Cellular Uptake in HASMCs Without Affecting Cytotoxicity

**Figure 6:**
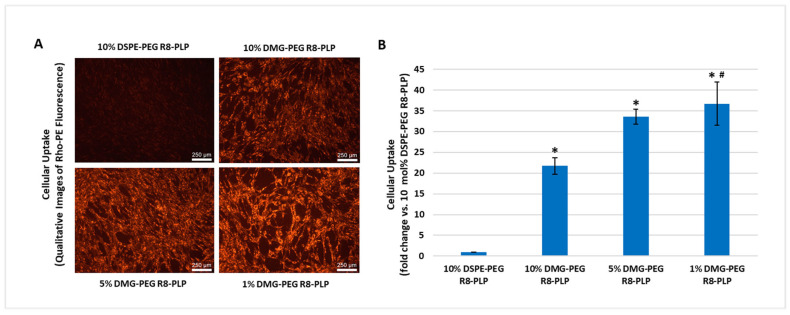
Effect of variable mol% DMG-PEG on R8-PLP cellular uptake. Based on (**A**) qualitative and (**B**) quantitative analysis, incorporation of DMG-PEG at all mol% increased cellular uptake compared to the DSPE-PEG control. * *p* < 0.05 vs. R8-PLP 10% DSPE-PEG, # *p* < 0.05 vs. R8-PLP 10% DMG-PEG, n = 3.

**Figure 7:**
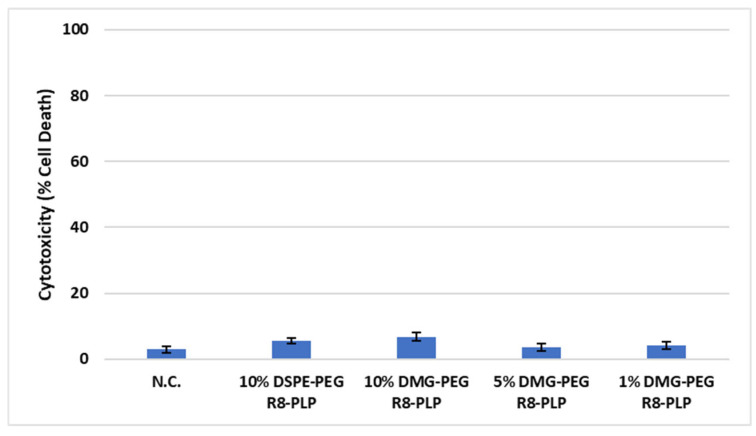
Effect of variable mol% DMG-PEG on R8-PLP induced cytotoxicity. Incorporation of DMG-PEG at all mol% did not alter cell viability compared to DSPE-PEG control or the non-treated negative controls (N.C.). *p* = NS vs. N.C., n = 3.

HASMCs treated with 50 uM total lipid for all DMG-PEG R8-PLPs demonstrated a significant increase in cellular uptake compared to equimolar DSPE-PEG R8-PLP treated controls (Figure 6, Figure S2). At 10, 5, and 1 mol% DMG-PEG incorporation, R8-PLPs demonstrated a 21.71 ± 1.94, 33.59 ± 1.74, and 36.70 ± 5.18-fold increase, respectively, compared to 10 mol% DSPE-PEG R8-PLP controls (* *p* < 0.05, n = 3). Decreasing to 1 mol% DMG-PEG incorporation further enhanced cellular uptake compared to 10 mol% DMG-PEG incorporation (# *p* < 0.05, n = 3). Furthermore, neither DSPE-PEG control formulations nor substitution with DMG-PEG at any mol% had a significant effect on cytotoxicity over baseline cell death in non-treated control cells (Figure 7).

### 3.7. Substitution of DMG-PEG for DSPE-PEG Did Not Significantly Affect R8-PLP-Mediated Gene Silencing In Vitro

**Figure 8:**
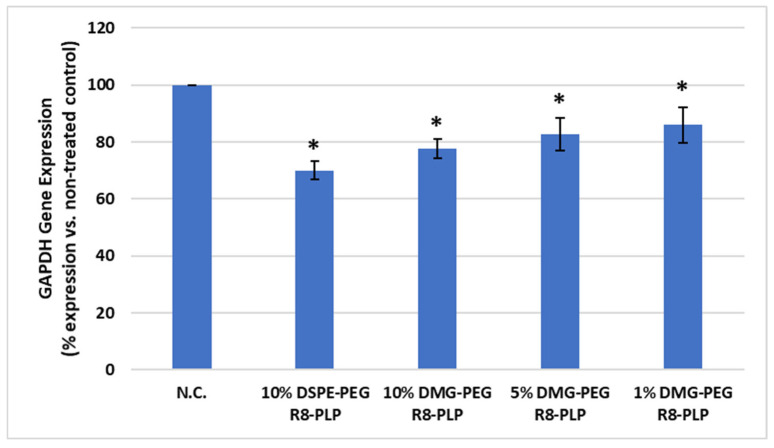
Effect of variable mol% DMG-PEG on R8-PLP mediated gene modulation in vitro. All DMG-PEG R8-PLP formulations demonstrated significant silencing compared to the non-treated control. (* *p* < 0.05 vs. N.C., n = 6). However, DMG-PEG R8-PLPs did not improve gene silencing compared to 10% DSPE-PEG R8-PLP.

HASMCs were treated with all R8-PLP formulations, each delivering 50 nM *GAPDH* siRNA. At 10, 5, and 1 mol% DMG-PEG incorporation, R8-PLP-mediated siRNA delivery resulted in significant gene silencing compared to the non-treated control (22.4 ± 0.05%, 17.2 ± 0.08%, and 14.0 ± 0.08% silencing, respectively, Figure 8, *p* < 0.05, n = 6). However, all DMG-PEG R8-PLP groups demonstrated less efficient gene silencing compared to siRNA delivery via the 10 mol% DSPE-PEG R8-PLP controls (30.0 ± 0.03% silencing).

## 4. Discussion

RNA therapeutics have gained significant attention in the last twenty years due to their potential to revolutionize modern medicine by attenuating diseases at the molecular level. RNA-based therapeutics have been developed against a variety of conditions including genetic disorders, infectious diseases, and cancers, with the successful transition of many to clinical trials. However, despite their potential, naked RNA molecules are unstable, cannot readily cross cell membranes or escape endosomes, and require delivery platforms to be effective [1,19]. Lipid-based nanoparticles represent the most extensively developed delivery platform for RNA therapeutics due to their biocompatibility and ability to protect RNA cargo from nuclease degradation while simultaneously facilitating cellular delivery [5,20]. LNPs are generally comprised of four components: bulk phospholipids, cholesterol for membrane rigidity, cell penetrating molecules (i.e., ionizable-cationic lipids, cell penetrating peptides, etc.), and PEG-lipid conjugates to improve LNP stability and circulation half-life [6,21].

PEG consists of a series of repeating ethylene oxide subunits that form a long hydrophilic chain [7]. The PEG chain is then conjugated to a lipophilic structure which embeds the molecule into the LNP membrane [7]. As PEG protrudes from the LNP surface, it creates a steric barrier. This barrier enhances LNP stability by preventing serum–protein interactions, inter-particle fusion, and reducing LNP clearance by the MPS [7,9,22]. Although PEG incorporation can enhance LNP stability and pharmacokinetics, it also creates two major challenges that can impede their efficacy: the PEG dilemma and anti-PEG antibodies.

The PEG dilemma is a paradox that refers to how PEG’s physical barrier simultaneously improves and impairs LNP efficacy [6,11,23]. While PEGylation increases LNP stability by providing a physical barrier to mitigate deleterious interactions with other entities, this barrier can also prevent membrane interactions necessary for internalization, specifically hindering target cell apposition and increasing endosomal entrapment [24,25]. Thus, PEGylated LNPs have demonstrated decreased drug delivery and reduced therapeutic effects.

Likewise, PEGylation can inhibit LNP efficacy by the accelerated blood clearance phenomenon [15,26,27]. Although free PEG is immunogenically inert [12,13], PEG bound to another entity can induce an immune response, resulting in anti-PEG antibody production [27,28]. Specifically, antibodies against PEG’s repeating ethylene oxide subunits are the most problematic, as these can be induced not only by LNPs but also various PEGylated therapeutics and commercial products through everyday routine exposures [29,30,31,32]. These cross-reactive antibodies, regardless of origin, can reduce LNP efficacy by stimulating the immune system to rapidly clear PEGylated LNPs from the body [27,32,33]. This is especially problematic for therapeutics that may require repeat dosing, as these secondary administrations may be cleared before effectively executing their function. Consequently, anti-PEG antibody prevalence limits the long-term use of PEGylated LNPs as they diminish stability, circulation, and overall efficacy. Despite these challenges, PEG moieties are still the most effective molecules for increasing LNP stability and favorable pharmacokinetics for clinical utilization [7]. Therefore, research efforts have explored methods to maintain PEG incorporation while reducing its negative effects.

One of the most promising strategies is to induce PEG dissociation by altering the length of the PEGylated lipid’s carbon tail [15]. In PEG-lipid moieties, the PEG component is large and hydrophilic, thus favoring aqueous environments [9,10]. Consequently, PEG’s retention time on the LNP is determined by the strength of its lipid anchor. Longer 18 carbon lipid tails occupy a greater surface area within the nanoparticle membrane, subsequently producing stronger hydrophobic forces that more readily anchor PEG to the particle [9,11]. Conversely, PEG-lipid moieties with short 14 carbon tails form weaker anchors, as their shallow embedding reduces the intermolecular forces within the nanoparticle membrane [9,11,16]. Thus, in aqueous, serum-like conditions PEG’s hydrophilicity can overcome the weak anchor, resulting in its spontaneous dissociation [16]. Findings consistently indicate that this PEG desorption associated with short lipid tails provides greater gene transfection compared to long-anchored PEG-lipid conjugates [6,10,16]. Specifically, the increased PEG dissociation frees up the LNP surface for target cell apposition, allowing efficient LNP internalization without negatively impacting LNP stability. Likewise, short-anchored PEG-lipids are advantageous because they reduce anti-PEG antibody induction. As these short anchors increase PEG’s desorption rate, it reduces the available bound PEG for immune activation and recognition [14,34]. Comparatively, long-anchored PEG-lipids, because of their reduced shedding rate, correlate to a higher immune response with greater anti-PEG induction [10,14]. Thus, utilizing a more rapidly dissociating short-anchored PEGylated lipid can circumvent the challenges associated with PEGylation.

Due to the potential for enhanced LNP efficacy and long-term immunogenic benefits of short-tailed PEGylated lipids, we investigated if the incorporation of DMG-PEG, a 14 carbon tailed PEGylated lipid utilized in the COVID-19 Moderna Spikevax vaccine, would enhance the efficacy of our R8-PLP platform without sacrificing preliminary in vitro CQAs, parameters that must be met before moving to pre-clinical in vivo models and as preliminary criteria toward eventual clinical translation. Our previously patented R8-PLP formulation incorporates 10 mol% DSPE-PEG for membrane stability and pharmacokinetics [17,18]. Here we substituted 10 mol% DMG-PEG in place of our standard 10 mol% DSPE-PEG. Additionally, we investigated 1 and 5 mol% DMG-PEG incorporation based on the literature suggesting that 14 carbon tailed PEGylated lipids demonstrate enhanced LNP efficacy at low mol% incorporation [10,14].

Substitution of DMG-PEG for DSPE-PEG resulted in subtle morphological differences in R8-PLPs, but no change in distinct LNP structural classification. Conventional TEM of R8-PLPs with 1 and 10 mol% DMG-PEG, as well as 10 mol% DSPE-PEG controls, revealed nanoscale, predominantly spherical particles for all three formulations, with amorphous internal contrast and no visible bilayer or multilamellar structures, consistent with LNP-type assemblies. Qualitative differences in particle compactness, electron density, and aggregation behavior were observed between formulations. However, given the inherent limitations of conventional TEM in resolving hydrated lipid nanostructures, quantitative in vitro CQAs were analyzed and served as the primary basis for nanoparticle characterization.

Because short-anchored PEG-lipids are known to increase desorption rates, it was necessary to ensure that this dissociative behavior would not impact R8-PLP CQAs at assembly or R8-PLP stability during storage. Ball et al. [35] found that incorporation of DMG-PEG maintained stable LNPs at refrigerator temperatures for up to 150 days in aqueous conditions. Thus, we hypothesized that DMG-PEG’s dissociation potential would not impact the assembly parameters or the storage stability of our R8-PLPs in PBS at refrigerator temperature, a condition readily available in clinical settings. For nanoparticles to be prospectively considered suitable for eventual clinical translation by the FDA, they must have a characterization profile addressing the following preliminary in vitro CQAs: mean particle size, particle size distribution, membrane zeta-potential, and encapsulation efficiency [36]. To be considered a nanomaterial and ensure prolonged blood circulation, LNPs must maintain an average size of less than 100 nm [13,37]. For FDA approval the size distribution of these nanoparticles must be relatively homogenous, with the general consensus for LNP development being PDI ≤ 0.3 to ensure nanoparticle monodispersity [38]. Moreover, nanoparticles are generally expected to have a near-neutral membrane zeta-potential (−10 mV to +10 mV) in order to minimize toxicity and immune activation, with zeta-potentials above +30 mV considered to be strongly cationic [39]. Likewise, for effective and scalable manufacturing, higher drug encapsulation is prioritized as it reduces waste and cost [5,40]. Here we demonstrated that at assembly all CQAs of all DMG-PEG R8-PLP formulations were comparable with the DSPE-PEG R8-PLP controls, as well as preliminary in vitro CQA guidelines, with sizes of <65 nm, PDI < 0.3, and EE% > 85%. Importantly, zeta-potentials of three of our four R8-PLP formulations were near-neutral at or below +10 mV, a preliminary parameter for pre-clinical progression. Only the 1 mol% DMG-PEG R8-PLP had a non-neutral charge, likely due to the decreased PEG content and subsequent diminished charge shielding. Likewise, all size, PDI, and EE% parameters were maintained in all formulation groups for up to 28 days when stored at refrigerator temperatures, demonstrating that LNPs retained their full siRNA drug load without any membrane degradation or particle aggregation.

To more thoroughly characterize the profiles and physiological behaviors of our DMG-PEG R8-PLP variants, in light of potential increased desorption, we evaluated LNP stability and drug release kinetics in serum. All DMG-PEG R8-PLPs, as well as DSPE-PEG R8-PLP controls, retained their full siRNA drug load and maintained baseline size and particle homogeneity for up to 7 days following storage in serum at 4 °C. These results indicate no membrane aggregation or degradation as a result of serum–protein interactions at refrigerator temperatures. To investigate R8-PLP siRNA release kinetics at physiological conditions, all formulations were incubated in 0% and 20% serum at 37 °C. In serum-free conditions, all R8-PLPs maintained their full siRNA load for up to 18 h. However, when incubated in 20% serum, all DMG-PEG R8-PLP formulations demonstrated significant and accelerated siRNA release by hour 6. In contrast, DSPE-PEG R8-PLP controls exhibited a more delayed release kinetic profile, taking up to 12 h to release comparable levels of siRNA cargo. Although these ex vivo release kinetics do not fully grasp the complexity of a physiological system, they do provide a simplified model to ascertain information regarding basic serum–protein interactions and membrane stabilization at controlled storage and physiological temperatures.

Due to the weak embedding of short carbon tailed PEGylated lipids, and their reduced steric barrier for membrane interactions, studies have consistently observed increased cellular uptake in serum-like conditions [41,42]. As such, here we investigated if DMG-PEG incorporation would enhance R8-PLP cellular uptake in HASMCs without deleterious effects on LNP-induced cytotoxicity. HASMCs were selected as our primary cell line due to their involvement in intimal hyperplasia induced restenosis pathogenesis, the ultimate clinical target of our delivery system. In fact, all DMG-PEG R8-PLPs demonstrated significantly enhanced cellular uptake over our standard DSPE-PEG R8-PLP formulation. This suggests that the short-anchored PEGylated lipids desorbed effectively and subsequently liberated the LNP surface for greater cellular membrane interaction. Notably, although DMG-PEG maintains the same hydrophilic PEG polymer as our DSPE-PEG constituent, its lipid component is a derivative of endogenous dimyristoyl phosphatidylcholine [43]. Despite its naturally occurring subunits, DMGs structure is not observed in lipid membranes, and DMG is considered a synthetic component. This necessitates evaluation of any potential contribution to lipid-induced cytotoxicity. As expected, the lipid burden of all R8-PLPs had no in vitro cytotoxic effect, demonstrating no notable increase in cell death over baseline cells with no LNP exposure.

According to the literature the dissociative nature of short-anchored PEG-lipids is not only associated with enhanced cellular uptake, some studies have also demonstrated their utilization for greater gene therapeutic efficacy compared to long-anchored PEG. In a study by Mui et al. [10], treatment with DMG-PEG LNPs loaded with factor VII targeted siRNA demonstrated significant silencing in an in vivo mouse model. Likewise, several studies have demonstrated this same therapeutic efficacy trend in vitro [16,41,42]. Here we assayed our DMG-PEG formulated R8-PLPs for efficacious silencing of *GAPDH* in HASMCs in vitro. Although our long-term goal for this LNP technology is modulation of disease relevant gene targets for vascular disease mitigation—primarily the matrix metalloproteinase (MMP) family—many of these targets are not constitutively expressed in healthy unstimulated HASMC cultures, and are only upregulated following vascular injury or during pathogenesis. Thus, these disease-related targets cannot be readily investigated in vitro. Instead, here we selected *GAPDH* as a highly expressed housekeeping gene as a proof-of-concept target due to its abundant expression and ease of efficacy evaluation. As expected, all DMG-PEG R8-PLPs resulted in significant *GAPDH* silencing over baseline expression of non-treated controls. Consistent with previous studies, 10 mol% DSPE-PEG R8-PLPs achieved statistically significant and biologically relevant silencing within the limitations of in vitro gene knockdown assays [17]. However, although substitution of DMG-PEG for DSPE-PEG significantly enhanced cellular uptake, this did not correlate to enhanced silencing efficacy compared to our standard DSPE-PEG R8-PLP formulation. We speculate that this discrepancy may reflect LNP endosomal entrapment due to undesirable STR-R8 shedding following DMG-PEG dissociation. The literature demonstrating improved LNP-mediated silencing efficacy with DMG-PEG consistently utilizes a bulk ionizable lipid to promote endosomal escape and improve cytosolic delivery. However, Chen et al. [44] found that the rapid loss of DMG-PEG in small ionizable LNPs correlated with early dissociation of the ionizable lipid during the early stages of endocytosis, limiting endosomal escape and silencing efficacy. Although our R8-PLP platform lacks this ionizable lipid component, it instead incorporates a lipopeptide moiety (STR-R8) to facilitate cell entry and endosomal escape. If Chen et al.’s observations are applicable to STR-R8, early dissociation of STR-R8 could potentially reduce its LNP surface availability and theoretically diminish its facilitation of endosomal escape. This could hypothetically reconcile the discrepancy between the DMG-PEG R8-PLPs increased cellular uptake and the lack of corresponding improvement in gene modulation.

Additionally, although there was no improvement in gene silencing efficiency compared to DSPE-PEG R8-PLPs, there may still be long-term benefits from the incorporation of DMG-PEG into our previously established R8-PLP formulation. One of the primary advantages for the utilization of DMG-PEG is the potential ability to attenuate PEG-related immune responses and the production of anti-PEG antibodies. Because DMG-PEG R8-PLP formulations did achieve significant silencing compared to the non-treated controls, they may in fact be applicable and potentially superior for clinical uses that require multiple or repeat administrations. Future in vivo studies should still investigate the benefits of incorporating DMG-PEG in our R8-PLP platform regarding immunogenicity and repeat administration efficacy. Specifically, if these short-anchored PEGylated formulations can achieve consistent silencing with multiple dose administration, their slightly diminished silencing efficiency compared to long-anchored PEGylated formulations may be negligible in the long-term utilization of these formulations, making them advantageous for our ultimate goal of clinical translation in chronic ongoing pathogenic processes.

## 5. Conclusions

Overall, the substitution of DMG-PEG for DSPE-PEG in our R8-PLP formulation resulted in the assembly of nanoparticles demonstrating all necessary preliminary in vitro CQAs for pre-clinical advancement and storage in realizable clinical environments. DMG-PEG R8-PLPs also demonstrated improved cellular uptake without any impact on cytotoxicity. However, DMG-PEG incorporation did not enhance gene silencing efficacy over our standard DSPE-PEG R8-PLP platform either at equi-mol% or at reduced PEGylation. Although this short-anchored PEG-lipid modification did not yield the results we expected, short-anchored PEG-lipid incorporation still provides a promising strategy to mitigate some of the challenges associated with PEG. For example, future studies could still investigate the benefits of incorporating DMG-PEG in our R8-PLP platform regarding long-term immunity and potential secondary administration in vivo. Specifically, if these short-anchored PEGylated formulations can achieve consistent silencing with multiple dose administration, their diminished silencing efficiency compared to long-anchored PEGylated formulations may be negligible in the long-term utilization of these formulations, making them advantageous for our ultimate goal of clinical translation in chronic ongoing pathogenic processes.

## Figures and Tables

**Table 1:** R8-PLP lipid formulation constituents.

Lipid Constituent	Acronym	Lipid Structure (Obtained from Avanti Polar Lipids and LifeTein LLC)
1,2-dioleoyl-sn-glycero-3-phosphocholine	DOPC	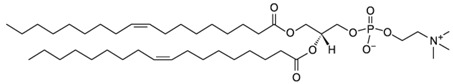
Cholesterol	Chol	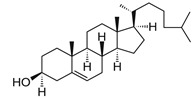
1,2-distearoyl-sn-glycero-3-phosphoethanolamine-N-[methoxy(polyethylene glycol)-2000]	DSPE-PEG	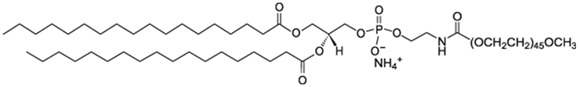
1,2-dimyristoyl-rac-glycero-3-[methoxy(polyethylene glycol)-2000]	DMG-PEG	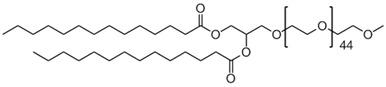
1,2-dipalmitoyl-sn-glycero-3- phosphoethanolamine-N-(lissamine rhodamine B sulfonyl)	Rho-PE	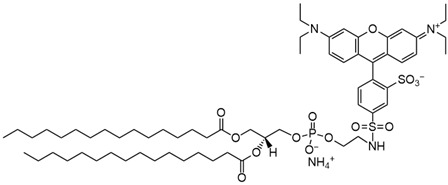
Stearylated octaarginine	STR-R8	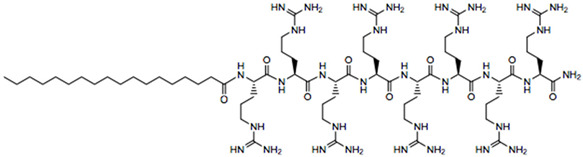

**Table 2:** Control and experimental R8-PLP formulation details.

	DOPC	Chol	DSPE-PEG	DMG-PEG	STR-R8
10 mol% DSPE-PEG R8-PLP	56 mol%	24 mol%	10 mol%	0 mol%	10 mol%
10 mol% DMG-PEG R8-PLP	56 mol%	24 mol%	0 mol%	10 mol%	10 mol%
5 mol% DMG-PEG R8-PLP	59.5 mol%	25.5 mol%	0 mol%	5 mol%	10 mol%
1 mol% DMG-PEG R8-PLP	62.3 mol%	26.7 mol%	0 mol%	1 mol%	10 mol%

**Table 3:** Size, homogeneity, and zeta-potential of R8-PLP lipid formulations.

R8-PLP mol% PEG	Size (nm)	PDI	Zeta-Potential (mV)
10% DMG-PEG	50.5 ± 0.8	0.12 ± 0.038	6.98 ± 0.302
5% DMG-PEG	50.5 ± 0.4	0.12 ± 0.009	10.41 ± 0.097
1% DMG-PEG	61.0 ± 2.1	0.19 ± 0.012	19.53 ± 0.842
10% DSPE-PEG	60.2 ± 2.4	0.25 ± 0.005	6.73 ± 0.298

## Data Availability

The original contributions presented in this study are included in the article. Further inquiries can be directed to the corresponding author.

## Supplementary Materials

The following supporting information can be downloaded at: https://www.mdpi.com/article/10.3390/biomedicines14051002/s1, Figure S1: Gaussian Distributions of all R8-PLPs comparing Day 0 distribution to Day 28 following storage in PBS at 4 °C; Figure S2: Brightfield HASMC Cell Culture Images. All brightfield images correspond to fluorescent images in Figure 6A.

## Abbreviations

The following abbreviations are used in this manuscript:

RNA	Ribonucleic Acid
RNAi	RNA Interference
LNP	Lipid Nanoparticle
PEG	Polyethylene Glycol
MPS	Mononuclear Phagocytic System
R8-PLP	Non-cationic LNP Platform
DSPE-PEG	1,2-distearoyl-sn-glycero-3-phosphoethanolamine-N-[methoxy(polyethylene glycol)-2000]
DMG-PEG	1,2-dimyristoyl-rac-glycero-3-[methoxy(polyethylene glycol)-2000]
CQA	Critical Quality Attributes
DOPC	1,2-dioleoyl-sn-glycero-3-phosphocholine
Chol	Cholesterol
Rho-PE	1,2-dipalmitoyl-sn-glycero-3-phosphoethanolamine-N-(lissamine rhodamine B sulfonyl)
STR-R8	Stearylated-Octaarginine
siRNA	Small Interfering RNA
PBS	Phosphate-Buffered Saline
EE%	Encapsulation Efficiency
PDI	Polydispersity Index
DLS	Dynamic Light Scattering
FBS	Fetal Bovine Serum
HASMC	Human Aortic Smooth Muscle Cells
DMEM	Dulbecco’s Modified Eagle Medium
FITC	Fluorescein Isothiocynate
qPCR	Quantitative Polymerase Chain Reaction
mRNA	Messenger RNA
TEM	Transmission Electron Microscopy
N.C.	Non-treated Negative Control
