# Supplementary material for: Effect of Short-Anchored PEGylated Lipids on Lipid Nanoparticle Characterization Profiles, Stability, and Efficacy
Source: Biomedicines. 2026 Apr 28;14(5):1002. doi: 10.3390/biomedicines14051002 (PMC13205063; doi:10.3390/biomedicines14051002)
Supplement: Supplementary file 1 [file biomedicines-14-01002-s001.zip › biomedicines-4215540-supplementary.pdf]

Supplemental Materials

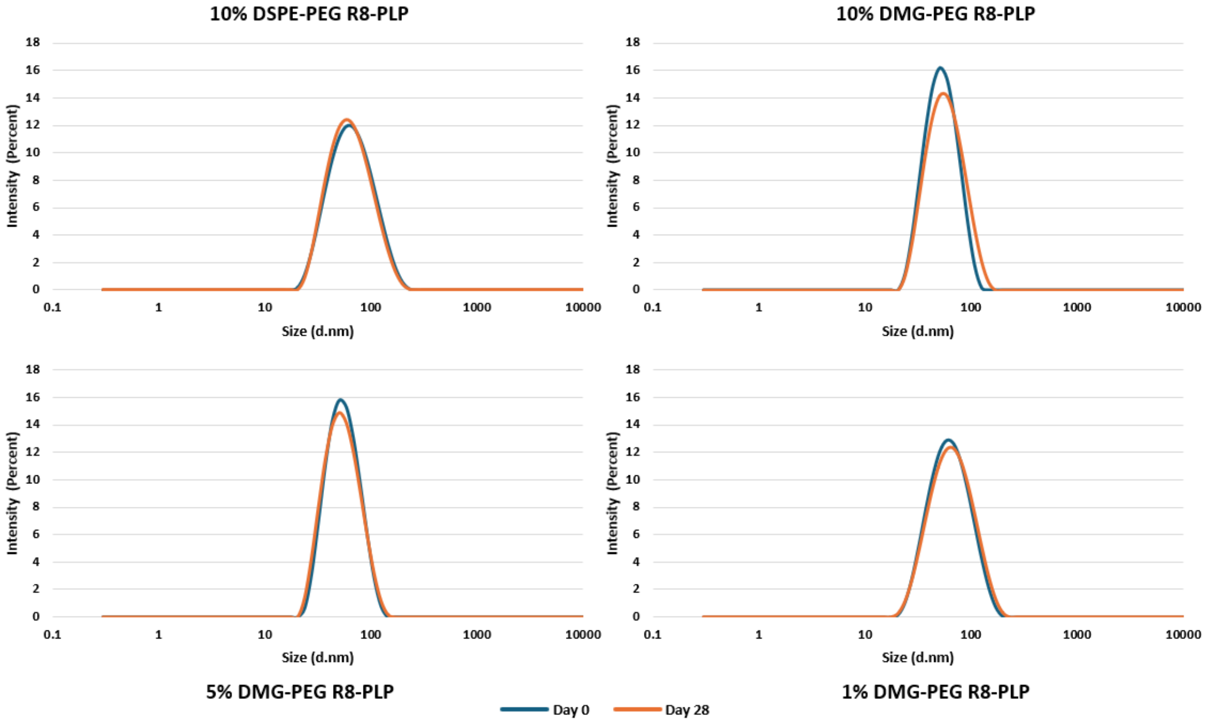

Figure S1. Gaussian Distributions of R8-PLPs comparing Day 0 distribution to Day 28 following storage in PBS at 4°C.

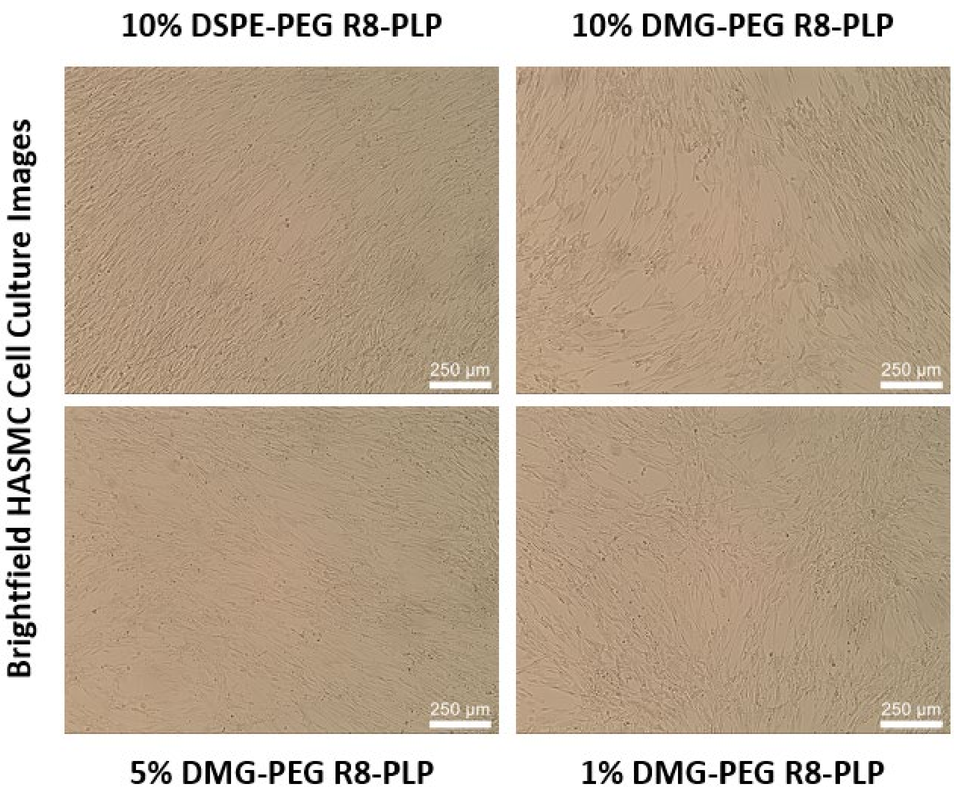

Figure S2. Brightfield HASMC Cell Culture Images. Brightfield images correspond to fluorescent microscopy images in Figure 6A.
